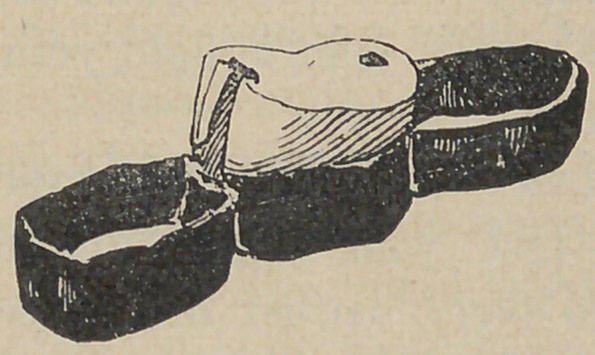# Prehistoric Dentistry—Bridge Work

**Published:** 1889-05

**Authors:** 


### Prehistoric Dentistry.—Bridge-Work.

The following from Dr. J. G. Van Marter, D.D.S., of Rome, Italy, in the main is self-explanatory. The accompanying illustration is a little larger than the specimen. It is made of pure gold; the bands seem to be welded rather than soldered as no solder appears at the joints. The outside bands embraced two natural teeth to support the piece, the central band or socket supported the substituted tooth, which is a natural human tooth, a bicuspid, turned one-fourth round on its axis. This was done probably to more completely fill the space, than the tooth would do placed in its natural position.

The following from Dr. Van Marter, accompanied the specimen.

My Dear Doctor:—I send you by this post a prehistoric tooth taken from an Etruscan tomb, lately opened not far from Rome on the lake of Valseno. It dates six hundred years before Christ. The skull it was taken from soon crumbled on exposure to the air and hence I am disappointed in not being able to send you this specimen of ancient work in situ."

This is the oldest denture that has ever come to the notice of the writer so far as memory now serves. It certainly antedates the work upon which patents have been issued and which is now a matter of so much solicitude to the profession.

## Figures and Tables

**Figure f1:**